# Liposome Drug Delivery System across Endothelial Plasma Membrane: Role of Distance between Endothelial Cells and Blood Flow Rate

**DOI:** 10.3390/molecules25081875

**Published:** 2020-04-18

**Authors:** Olga E. Glukhova

**Affiliations:** 1 Department of Physics, Saratov State University, Astrakhanskaya Street 83, 410012 Saratov, Russia; glukhovaoe@info.sgu.ru; Tel.: +7-8452-514562; 2 Laboratory of Biomedical Nanotechnology, I.M. Sechenov First Moscow State Medical University, Bolshaya Pirogovskaya Street 2-4, 119991 Moscow, Russia

**Keywords:** liposome, in silico method, coarse-grained molecular dynamics, endothelial cells, intercellular gaps, membrane bilayer, blood flow

## Abstract

This paper discusses specific features of the interactions of small-diameter liposomes with the cytoplasmic membrane of endothelial cells using in silico methods. The movement pattern of the liposomal drug delivery system was modeled in accordance with the conditions of the near-wall layer of blood flow. Our simulation results show that the liposomes can become stuck in the intercellular gaps and even break down when the gap is reduced. Liposomes stuck in the gaps are capable of withstanding a shell deformation of ~15% with an increase in liposome energy by 26%. Critical deformation of the membrane gives an impetus to drug release from the liposome outward. We found that the liposomes moving in the near-wall layer of blood flow inevitably stick to the membrane. Liposome sticking on the membrane is accompanied by its gradual splicing with the membrane bilayer. This leads to a gradual drug release inside the cell.

## 1. Introduction

Advanced drug delivery molecular systems have numerous applications in various fields, especially in bio- and nanotechnologies [1,2,3,4]. About 50 years ago, Bangham pioneered this field by observing that phospholipids dispersed in water can build a new form of vesicular structure consisting of lipid bilayers [5]. These structures, so-called liposomes, are biodegradable and biocompatible self-forming spherical lipid bilayer vesicles. They have become effective molecular nanomedicine systems reaching clinical application [6,7]. As is known, liposomes are spherical vesicles that have a lipid bilayer shell enclosing an aqueous core. Such a structure provides the possibility of carrying a broad spectrum of drugs. The topic of using liposomes as scaffolds for drug delivery is being actively discussed and developed. At present, both active and passive targeting are known. Active targeting is provided by the modification of the natural distribution patterns of liposomes [8,9]. One way of achieving this is by attaching ligands to liposomes. The attached ligands recognize and bind the liposomes to specific molecular cues on the surface of target cells [10,11,12]. In the case of passive targeting, liposome delivery is guided by the liposome’s natural distribution patterns [13,14]. Currently, higher-level complexity “bulk-scale” liposomal systems are also being investigated. These liposomal systems are designed for delivery of encapsulated drugs to therapeutic sites. Special attention has been given to liposome biophysics and the interaction of liposome membranes with encapsulated cargo. Some detailed reviews are devoted to this topic [15,16,17]. The wide prospects for the use of liposomes are confirmed by the active search for inexpensive and effective technologies for their large-scale production [18].

One of the main applications of liposomes is drug delivery directly to the tumor [19,20,21,22,23,24,25,26,27,28,29,30,31]. For this purpose, ligand-conjugated liposomes are used. These liposomal systems are designed to target receptors on tumor cells to decrease the drugs’ side effects. Liposomes of this type penetrate through the gaps between endothelial cells and accumulate in the microenvironment of the tumor. Another important area of liposome application is drug delivery to the brain. Today, photodynamic (PD) therapy is a very promising method for the treatment of brain glioma. To increase the quality of a personalized photodynamic effect on a glioma, the use of liposomes with photosensitizers to enhance the local antitumor effect is being planned [32]. In this case, the interaction of liposomes with endothelial cells is also very important.

Many papers have been devoted to the study of the interaction of liposomes with endothelial cells of different vessels [29,30,31,32,33]. However, the patterns of interactions of liposomes with the endothelial cell membrane and with the intercellular gap are not well understood. Today, several variants are considered: (1) penetration directly through the membrane; (2) capture of the liposomes by endosome vesicles; and (3) diffusion through disturbed tight junctions (they can be more than 7 nm in the nidi of pathologies like glioma). An experimental study of these processes is practically impossible, because they occur at the atomic and molecular level for several nanoseconds. In a natural experiment, it is impossible to determine the energy characteristics of the interaction of liposomes with the cell membrane (adhesion energy, cohesive energy of liposomes to each other, elastic energy of the liposome during contact with two cells in the gap space). However, such parameters and the mechanism of liposome penetration can be investigated in silico as a result of numerical experiments. There are very few similar works, because the molecular dynamic modeling of such macromolecular systems and their processes requires large computational resources, making it sometimes unachievable. In recent papers, the structure of modified liposomes and their behavior in aqueous medium have been studied in silico [27,34,35]. There are only a few computer simulations of the interaction of lipid macromolecules with the cell membrane. Important questions remain open about effect of size, surface charge, and ligand arrangement on the macromolecule surface and permeation across cell membranes [36]. While computational simulations are contributing to significant progress in this area, some challenges remain, namely regarding the behavior of macromolecules and liposomes on the cell membrane, as well as the multi-scale nature of nano–bio interactions. These interactions can be evaluated using coarse-grained molecular dynamics (CGMD) simulations. Recent investigations using the MARTINI force field revealed the patterns of interactions between different nanoparticles and lipid membranes [37,38,39,40,41,42,43]. Molecular modeling approaches have shown to be a valuable tool for the nano–bio interface, providing qualitative results in good agreement with experiments [35,44,45,46,47].

In this paper, the specific features of the interaction of small-diameter liposomes moving in the blood flow with the cytoplasmic membrane of the endothelial cell and disturbed tight junctions were investigated in silico. The mechanism of liposome penetration through the cytoplasmic membrane is also discussed.

## 2. Computational Details

All molecular dynamics simulations were performed with the GROMACS software package version 5.0.7 [48] using the MARTINI force field version 2.2 [49]. A Noze–Hoover thermostat [50] was used to control the temperature. The leapfrog algorithm was used to integrate the equations of motion. Integration step was 0.001 ps.

**Figure 1:**
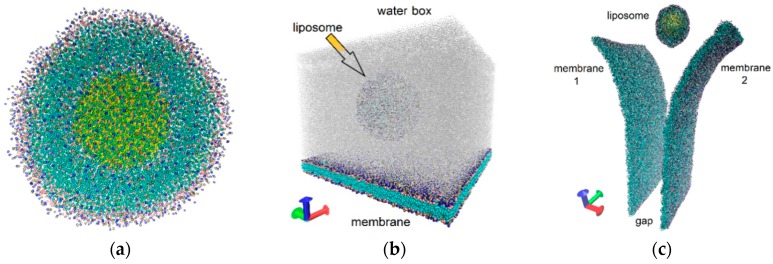
Coarse-grained (CG) models: (**a**) liposome; (**b**) liposome over the membrane; (**c**) liposome that penetrates into the gap between the membranes of two adjacent endothelial cells.

The liposome was modeled in the form of a sphere formed by a phospholipid bilayer of cholesterol (CHOL) molecules and dipalmitoylphosphatidylcholine (DPPC) molecules with a water droplet inside the cavity. The number of DPPC molecules was 78%, and number of CHOL molecules was 22% of the total number of molecules forming the vesicle, i.e., the DPPC/CHOL ratio was about 4:1. The water droplet simulated a drug that was carried by a liposome. A CG model of a small liposome with a diameter of about 20.6 nm was constructed to perform long-time numerical experiments that require large computational resources. The bilayer included 2240 CG molecules of DPPC and 642 CG molecules of CHOL, that is, it consisted of 34,584 grains. Figure 1a shows the liposome. The thickness of the bilayer liposome shell was 10 nm. The hydrophobic polar phospholipid heads formed the outer and inner layers of the shell; the hydrophilic phospholipid parts and cholesterol were located between them. The water droplet was formed by 3000 grains of water (they are shown in yellow in the figure). Each grain contained four molecules, so the whole droplet was formed by 12,000 H_2_O molecules. The diameter of the water droplet was 10.6 nm. In general, the coarse-grained model of the liposome included only 37,584 grains. The equilibrium structure of the liposome was obtained at a normal pressure and a temperature of 310 K.

The model of the endothelial cell membrane was constructed as a fragment of DPPC bilayers. The periodic box of 50 × 50 nm with translation in the X- and Y-directions (see Figure 1b) was generated to simulate the interactions of the liposome with the endothelial cell membrane. The bilayer included 8450 DPPC molecules; its CG model included 101,400 grains. The thickness of the layer was 5–5.5 nm. The equilibrium structure of the membrane was formed at a normal pressure and a temperature of 310 K.

Two membrane fragments of neighboring cells were constructed for a model of the gap between neighboring endothelial cells. As in the previous case, the membranes were formed by the DPPC bilayers in a periodic box with translation in the Y-direction (see Figure 1c). The shape of the bilayers reproduced the bend of the cell edge, reflecting the uneven shape of the gap. The box sizes were 72 nm in the X-direction (taking into account the curvature) and 50.1 nm in the Y-direction. The thickness of the bilayer was 5–6 nm. The size of the gap between the membranes varied from 20 to 22 nm, being slightly smaller and larger than the diameter of the liposome, respectively. The CG model included 521,376 grains and 43,448 CG molecules of DPPC. The equilibrium structure was obtained under the same conditions as the membrane.

## 3. Results and Discussion

**Figure 2:**
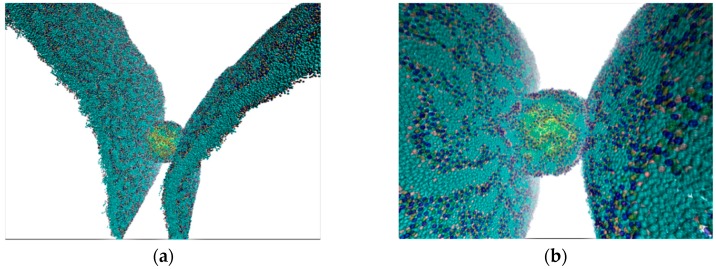
The model of liposome contact with endothelial cell membranes in the gap: (**a**) sticking of the liposome; (**b**) deformation of the liposome upon sticking. The water molecules are not shown; the water droplet inside the liposome is shown in yellow.

First of all, we investigated the mechanical elasticity of the liposome during the movement in the gap between endothelial cells. The distance between endothelial cells can vary greatly and increase from 10 nm to 100 nm and more in the case of a tumor [26]. On the other hand, the gap between cells varies depending on the type of vessel and its location in the human body, as well as on external factors. It is known that stress and other negative effects lead to vasoconstriction, that is, to a decrease in the gap between endothelial cells. The question arises: What mechanical loads can liposomes withstand? To answer this question, we conducted numerical experiments, when the liposome was compressed from the side of the endothelial cell walls during the movement between cells. The model of liposome contact with endothelial cell membranes in the gap is shown in Figure 2. The liposome moved along with water at a blood flow velocity of 0.1–1 m/s [51]. Water molecules were taken in the form of grains of four H_2_O molecules as in the case of the water droplet inside the liposome. Temperature was kept constant at 310 K. The viscosity of blood flow was taken equal to the viscosity of water of 0.001 Pa·s [52]. The most interesting results were obtained for models with a gap whose size was smaller than the diameter of the liposome. In the case where the diameter of the liposome was only ~0.5 nm smaller than the gap size, the liposome got stuck between walls, as shown in Figure 2a. Figure 2b clearly shows the deformation of the liposome shell. For clarity, all objects are transparent in Figure 2, so one can see well a water droplet inside the liposome. This figure also shows that the liposome remains unharmed under such a deformation and does not open, that is, it does not release the drug. After 20 simulations of the movement and sticking of the liposome in the gap, we found out that the liposome did not open in any case. Thus, an external mechanical action, for example, narrowing the gap, is necessary to open the liposome and release the water droplet imitating a drug.

**Figure 3:**
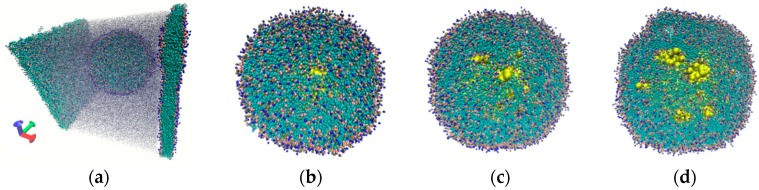
Simulation of narrowing the gap between cells: (**a**) a liposome in water between cell membranes (water is shown in gray); (**b**) liposome structure with an increase in diameter by 15%; (**c**) liposome structure with an increase in diameter by 23%; (**d**) liposome structure with an increase in diameter by 56%. The internal content of the liposome is marked in yellow.

Next, we simulated the narrowing of the gap and investigated the elasticity of the liposome. The simulation was carried out for the same liposome in water. To simulate narrowing the gap between cells, we used a model that was a box with two walls which were formed by two fragments of cell membranes, as shown in Figure 3a. The thickness of the phospholipid bilayer membranes was 5 nm. At the initial moment, the sizes of the box were 40 × 31 × 31 nm and the number of water molecules was 216,586. The simulation of narrowing the gap was carried out by compressing the box along the X-axis as a result of the simultaneous movement of the membranes towards each other. The compression velocity varied from 0.1 to 1 m/s. Results of 40 numerical experiments showed that the maximum deformation of the liposome was 14%–15%, regardless of the compression velocity (within these limits). This was the ultimate deformation for the liposome shell with a diameter of 20.6 nm filled with water. Starting at a value of 15%, the integrity of the liposome shell was disturbed and the internal filling could be released. Such a critical moment is shown in Figure 3b. It can be seen that, at that moment, the first pores opened in the liposome shell, and the water contained within the liposome was released outward. With further compression, the number of pores in the shell increased. For example, when the liposome shell was deformed by 20–23%, the number of pores became larger by several times, as shown in Figure 3c. When deformation exceeded 50%, the pores in the shell turned into large gaps, through which the contents of the liposome freely poured out. This situation is shown in Figure 3d.

**Figure 4:**
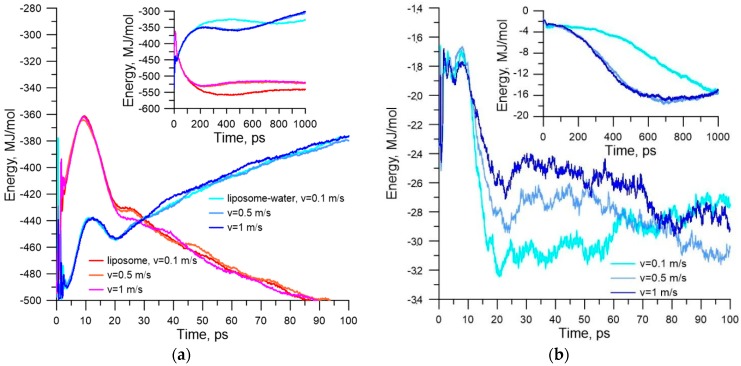
Energy of the liposome at different compression velocities: (**a**) energy of the liposome shell (red, pink, and orange curves) and energy of interaction of the liposome with the external water environment (dark blue and light blue curves); (**b**) energy of interaction of the liposome shell with the internal water droplet.

The energy profile of the liposome shell during its compression is shown in Figure 4a at three values of the compression velocity of 0.1, 0.5, and 1 m/s (red, pink, and orange curves, respectively). In all cases, compression was performed up to 70% in the diameter of the liposome. This is the limiting value of deformation when the liposome becomes a bilayer and the water droplet completely dissolves in the environment. Moreover, this result was achieved regardless of the compression velocity. As expected, the most significant changes in the energy were observed at the first moments of compression. The energy profile clearly shows a maximum in the first 8–10 ps, when the energy of the liposome shell increased by 26–27% in reaching the critical deformation of 14%–15%. At that moment, the external pressure of the water environment (dark blue and light blue curves in Figure 4a) and the internal pressure of the water droplet (curves in Figure 4b) also reached a maximum. As a consequence, this led to the liposome shell destruction. According to our calculations, the liposome shell can withstand a maximum pressure of 95 bar. After passing through the critical point of deformation, the energy of the liposome shell decreases sharply, as well as the energy of its interaction with the internal water droplet and the external environment. Further compression is accompanied by a decrease in the energy of the shell and the energy of interaction with the water droplet (internal medium) of the liposome, as is shown in the inserts of Figure 4. At the same time, the energy of interaction of the liposome shell disintegrating into DPPC bilayers with the water environment gradually increases, since the bilayers become part of the environment.

Another important phenomenon is the interaction of the liposome carrying the drug with the endothelial cell membrane during movement in blood flow. To simulate this phenomenon, we constructed a periodic box, as mentioned above, with a size of 50 × 50 nm. The cell membrane layer was located in a box in the XY plane. The liposome was located above the cell membrane layer. The box was filled with 676,169 water molecules. The CG model of the box containing 815,154 large grains is shown in Figure 1b (water molecules are marked in gray). The movement of blood flow was simulated along the X-axis.

**Figure 5:**
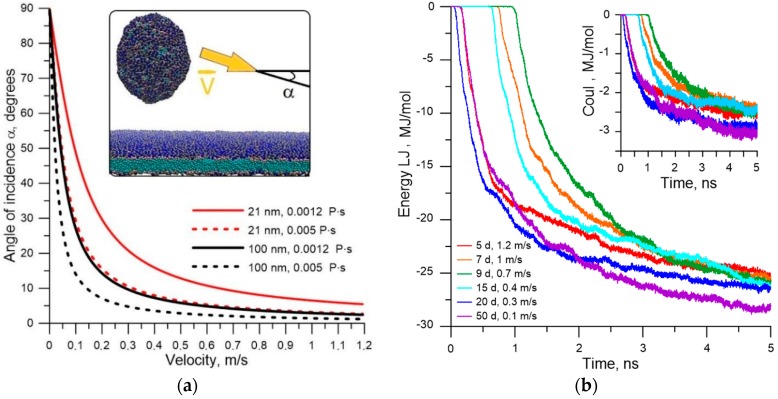
Model for simulating the impact and contact of the liposome with the cell membrane: (**a**) distribution of incidence angles of the liposome on the membrane depending on the velocity of blood flow for two liposomes with a diameter of 21 nm and 100 nm, and for two cases of medium viscosity; (**b**) energy of the van der Waals interaction of the liposome with the membrane and electrostatic interaction energy (insert in the plot).

The movement of the liposome in blood flow is regulated by two factors, namely flow velocity and Brownian motion. Using the Stokes–Einstein equation, one can describe the displacement of any nanoparticle or molecule in a plane perpendicular to the flow velocity vector (in the YZ plane) by the following expression:

(1) $${\langle x,y\rangle }^{2}=4\frac{k_{B}T}{3\pi d\eta }t$$

where *d* is the particle diameter, *η* is the coefficient of the medium viscosity, *t* is the time during which the particle is displaced, *k_B_* is the Boltzmann constant, and *T* is the absolute value of the medium temperature. From the relationship between velocity of displacement of the liposome to the membrane and velocity of blood flow along the membrane, one can obtain the distribution of incidence angles of the liposome on the cell membrane. Figure 5a shows the calculated distributions for liposomes with diameters of 21 and 100 nm at T = 310 K and viscosity values of 0.0012 Pa·s and 0.005 Pa·s [52]. As is known, the wall layer of blood flow has a lower viscosity, since this part of the flow is plasma. The velocities vary from a minimum value of 0.01 m/s [53], as in the capillaries, to a maximum value of 1.2 m/s [54], as in the large aorta. As can be seen from the plots in Figure 5a, the incidence angle of liposomes α lies in the range 0 < α < 15° for the most typical velocity range of 0.5–1.2 m/s (this velocity range is typical for the majority of middle and large vessels). Both small-diameter (21 nm) liposomes and liposomes with a diameter of 100 nm fit into this range. Since the wall layer of blood flow is represented by plasma, it is possible to consider water as the environment during the modeling of the contact of the liposome with the endothelial cell membrane. The viscosity coefficient of the water (0.0012 Pa·s) is close to the plasma viscosity.

Next, we simulated the interaction of the liposome with the membrane. The movement of the liposome with a diameter of 20.6 nm filled with water was modeled at velocities of near-wall blood flow of 0.1–1.2 m/s. According to Equation (1), the velocity of the liposome displacement in the YZ plane was 0.113 m/s in a medium with a viscosity of 0.0012 Pa·s at T = 310 K. This value was taken as the v_z_—component of the velocity. The v_x_-component was calculated taking into account the possible values of the incidence angle in Figure 5a. The velocity of the water was assumed to be equal to the blood flow velocity. The following patterns were obtained based on the results of a large number of numerical simulations.

First, the liposome shifts to the vessel wall and sticks to the endothelial cell membrane regardless of the blood flow velocity. This is due to the fact that the kinetic energy of the liposome is very small and is equal to 0.001–0.1 eV (at different velocities), and the van der Waals attraction from the membrane side is very strong. Therefore, the liposome is accelerated by van der Waals attraction and attracted by the membrane upon reaching a distance of ~0.8 nm regardless of the distance from the membrane and the flow velocity. Figure 5b shows the energy profiles of the Lennard-Jones potential (Energy LJ); the inset in the figure shows the energy curves of the Coulomb interaction (Coul). The presented curves differ in the incidence angle of the liposome, measured in degrees, and in the flow velocity. Three curves (green, orange, and blue) represent cases where the liposomes are removed at a distance of 3–4 nm from the membrane; therefore, the Lennard-Jones and Coulomb potentials remain zero within 1–1.5 ns. The other three curves show cases where the liposome starts to move immediately in the wall layer of 0.8–1 nm thick, when the van der Waals attraction already appears.

**Figure 6:**
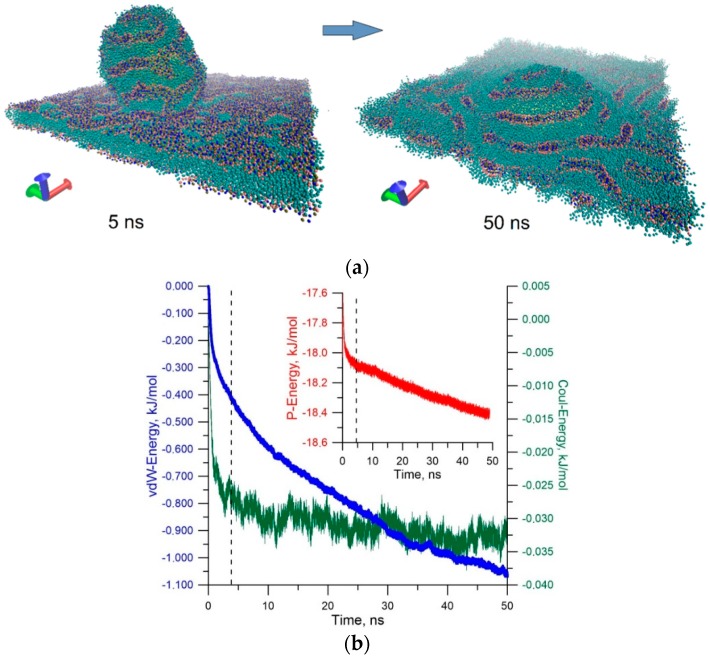
Model for simulating the impact and contact of the liposome with the cell membrane: (**a**) distribution of incidence angles of the liposome on the membrane depending on the velocity of blood flow for two liposomes with a diameter of 21 nm and 100 nm, and for two cases of medium viscosity; (**b**) energy of the van der Waals interaction of the liposome with the membrane and electrostatic interaction energy (insert in the plot).

Second, the sticking of the liposome is largely determined by the van der Waals attraction energy. The electrostatic sticking is 10 times weaker in energy in comparison with the van der Waals attraction energy. The energy of contact with the membrane is 27–30 MJ/mol (the sum of Lennard-Jones and Coulomb potentials). This leads to an insurmountable potential barrier for liposome detachment. We can assume that only a larger massive object could detach the liposome from the membrane. To answer the question about the further behavior of the stuck liposome, long-time calculations were carried out under various initial conditions of contact between liposome and membrane. Figure 6a shows stages of sticking liposome (snapshots of molecular dynamic modeling), and Figure 6b shows plots of energy of the van der Waals (vdW-Energy) and electrostatic interactions (Coul-Energy) between liposome and membrane, as well as the plot of the potential energy of the liposome–membrane complex (P-Energy). The plots in Figure 6b are the averaged result of 20 numerical simulations of collision and subsequent relaxation of the liposome–membrane complex at a blood flow velocity of 0.5 m/s.

Snapshots in Figure 6a clearly demonstrate a dramatic change in the distribution of the polar heads of the phospholipids of the liposome and membrane when they are spliced. Before contact with the bilayer surface, the distribution of the hydrophobic part of the bilayer is almost ideal. When the liposome makes contact with the membrane, the distribution of the hydrophobic part changes sharply already in the first nanoseconds (snapshot of 5 ns). In subsequent tens of nanoseconds, the membrane surface looks disturbed (snapshot of 50 ns). This is due to the natural reaction of the equilibrium structure of the membrane to external action from the liposome. Gradually, the membrane response is smoothed, which is well illustrated by the plots in Figure 6b. The dotted line indicates the moment of contact of the liposome with the membrane. Up to this moment, the liposome was attracted by the membrane, as evidenced by a reduction in the van der Waals energy. Furthermore, the electrostatic attraction is also observed. Then, there is a long period of relaxation of the liposome–membrane complex. This period is accompanied by a reduction in the potential energy and energy of the van der Waals interaction. During this period, the liposome is almost immersed in the membrane layer, so the van der Waals energy and potential energy decrease. This suggests that the splicing of the liposome with the membrane is advantageous in energy.

## 4. Conclusions

An in silico study of the behavior of a liposome with a diameter of ~21 nm during contacts with the endothelial cell membrane and an intercellular gap was conducted. It was shown that such liposomes containing drugs are able to withstand a shell deformation of 15% with an increase in liposome energy by 26%. With further mechanical action, the liposome membrane begins to disintegrate irreversibly with the release of internal content. The released drug remains in the intercellular gap and probably further penetrates under the layer of endothelial cells. On the other hand, the obtained data open up new possibilities for finding ways to open liposomes. An increase in the energy of the liposome by 26% or more ensures its opening. This can be achieved by heating the liposome, for example, by laser radiation, or by external mechanical action, for example, by ultrasound. The conclusions reached about the mechanical properties correlate with the well-known experimental studies of the elasticity of the liposome’s phospholipid shell. Some of the most significant works [55,56,57] demonstrate that liposomes from DPPC molecules exhibit greater compressive elasticity than liposomes from dioleoylphosphatidylcholine (DOPC). It was shown that a force of ~15 nN is required to compress by 7% the empty liposome from DPPC with a diameter of 4 μm. Further compression leads to a sharp increase in the required force. In our case, the liposome is filled with water, so it requires more force to compress. Therefore, a deformation of 15% requires a force of ~75 nN.

The simulation of the liposome movement in the blood flow, in particular in the flow of the near-wall plasma layer, showed the following results: (1) as a result of Brownian motion, the liposome inevitably shifts to the vessel wall and sticks to the cell membrane and (2) the energy of adhesion of the liposome to the membrane is large enough, so liposome bouncing and liposome rolling are not observed. The established fact that the liposome buries itself in the bilayer after sticking is very interesting. Consequently, the further fate of the liposome drug is determined by the fate of the cytoplasmic membrane. For example, in the case of the liposome shell fusion with the cell membrane, the drug inside the cell is more easily released due to the action of enzymes (esterases).

However, there is still the question about possible transcytosis in the case where the membrane and liposomes do not merge, and the liposome consists entirely of the endosome, i.e., surrounded by an additional membrane. This inhibits the release of the drug. Perhaps such an endosome will pass through the entire cytoplasm and come out from the opposite side of the cell.
